# Multiple Sclerosis and CCSVI: A Population-Based Case Control Study

**DOI:** 10.1371/journal.pone.0041227

**Published:** 2012-08-03

**Authors:** Francesco Patti, Alessandra Nicoletti, Carmela Leone, Silvia Messina, Emanuele D’Amico, Salvatore Lo Fermo, Vincenza Paradisi, Elisa Bruno, Graziella Quattrocchi, Pierfrancesco Veroux, Luigi Di Pino, Luca Costanzo, Mario Zappia

**Affiliations:** 1 Department DANA GF Ingrassia, Section of Neurosciences, University of Catania, Catania, Italy; 2 FIMG (Italian Federation of General Medicine), Catania Municipality Section, Catania, Italy; 3 Department of Vascular Surgery, University of Catania, Catania, Italy; 4 Department of Cardiology, University of Catania, Catania, Italy; Johannes Gutenberg University of Mainz, Germany

## Abstract

**Background:**

Chronic cerebrospinal venous insufficiency (CCSVI) has been associated to multiple sclerosis (MS).

**Objective:**

To evaluate the possible association between CCSVI and MS, using a population-based control design.

**Methods:**

A random cohort of 148 incident MS patients were enrolled in the study. We have also studied 20 patients with clinically isolated syndrome (CIS), 40 patients with other neurological diseases (OND), and 172 healthy controls. Transcranial (TCC) and Echo Color Doppler (ECD) were carried out in 380 subjects. A subject was considered CCSVI positive if ≥2 venous hemodynamic criteria were fulfilled.

**Results:**

CCSVI was present in 28 (18.9%) of the MS patients, in 2 (10%) of CIS patients, in 11 (6.4%) of the controls, and in 2 (5%) of the OND patients. A significant association between MS and CCSVI was found with an odds ratio of 3.41 (95% confidence interval 1.63–7.13; p = 0.001). CCSVI was significantly more frequent among MS subjects with a disease duration longer than 144 months (26.1% versus 12.6% of patients with duration shorter than 144 months; p = 0.03) and among patients with secondary progressive (SP) and primary progressive (PP) forms (30.2% and 29.4, respectively) than in patients with relapsing remitting (RR) MS (14.3%). A stronger association was found considering SP and PP forms (age adjusted OR = 4.7; 95% CI 1.83–12.0, p = 0.001); the association was weaker with the RR patients (age adjusted OR = 2.58; 95%CI 1.12–5.92; p = 0.02) or not significant in CIS group (age adjusted OR = 2.04; 95%CI 0.40–10.3; p = 0.4).

**Conclusions:**

A higher frequency of CCSVI has been found in MS patients; it was more evident in patients with advanced MS, suggesting that CCSVI could be related to MS disability.

## Introduction

Multiple sclerosis (MS) was firstly described by Charcot in 1868; since that it was known that plaques in MS are venocentric [1]. Post mortem studies and magnetic resonance (MR) venography demonstrated that veins could also be dilated and split longitudinally MS lesions [2]–[4]. Furthermore the presence of fibrin cuffs, perivenous iron deposits and iron laiden macrophages at histological examination of involved veins [5], [6] represented the rationale to investigate the cerebral venous drainage as a possible mechanism related to increased iron deposition in MS plaques and to the etiology of MS itself [7], and suggesting the concept that chronic cerebrospinal venous insufficiency (CCSVI) could have a causative role in MS [7]. More in details, venous drainage from brain and spine could be very frequently impaired in MS patients but not in control subjects [7], [8]. Moreover, it was described that Echo Color Doppler (ECD), Transcranial (TCC) sonography and other techniques were capable of showing abnormalities of cerebral and spine venous return [8]–[12]. These abnormalities and the existence of possible venous outflow abnormalities at extracranial level could be responsible for the high frequency of inversion of the physiological flow direction [10]. Nevertheless, successive recent studies have questioned the existence of CCSVI in MS [13]–[15]. A meta-analysis performed on studies that reported the frequency of CCSVI among patients with and those without MS showed a positive association between CCSVI and MS [16], but it was outlined that poor reporting of the success of blinding and marked heterogeneity among the studies precluded definitive conclusions [16].

In order to produce stronger evidence, we carried out a population case-control study to assess the possible association between CCSVI and MS. The presence of CCSVI was also evaluated in patients suffering either from clinically isolated syndrome (CIS), suggestive of demyelinating diseases, or from other neurological diseases (OND).

## Methods

### Ethics Statement

The study was approved by the two different local ethical committees (Azienda Universitaria-Ospedaliera Policlinico Vittorio Emanuele di Catania and Ethical Committe of the Azienda Sanitaria Locale 3 of Catania) and patients and controls were enrolled only after they signed the informed consent.

### Study Population

The survey was carried out in Catania, Sicily. Its official population in 2001, date of the last official census, was 313,110 inhabitants (165,065 women and 148,045 men). Since immigrants from other countries represent only 1% of the entire province of Catania (census 2001), our population can be considered ethnically stable.

Epidemiological surveys to determine the prevalence and temporal trend of MS in the city of Catania from 1975 to 2004 have been previously carried out [17], [18]. From 1 January 1975 to 31 December 2004, 367 MS patients resident in the study area had had the onset of disease. We considered as incident cases all patients who fulfilled Poser’s criteria for clinically definite MS (CDMS), laboratory supported definite MS (LSDMS), clinically probable MS (CPMS), and laboratory-supported probable MS (LSPMS) [19]. The clinical course of MS was assigned according specific criteria [20]. From this well defined incident cohort, MS patients were randomly selected using a computer generated list of random numbers and they were invited to participate at the present study.

At least one healthy control subject per each case, group matched by age and sex, were enrolled from the general population of Catania using a multistage sampling methods. In particular from the ten municipalities of the city of Catania we selected one or more General Practitioners (GPs). Control subjects, not affected by neurological disorders, were randomly selected from every GPs’ cabinet list. In order to exclude the presence of neurological disorders among controls we used a validated screening instrument already adopted in the Sicilian neuroepidemiologic surveys [21]. Controls who resulted positive at the screening underwent a complete neurological examination.

Exclusion criteria for both patients and controls were preexisting medical conditions known to be associated with brain pathology (e.g., cerebrovascular disease, positive history of alcohol abuse), history of cerebral congenital malformations, thrombosis of jugular veins, deep venous thrombosis, central venous catheter, head and neck surgery, transient global amnesia, vasculitis and pregnancy. Patients with MS were excluded according to the presence of relapses and steroid treatment in the 30 days preceding study entry.

Other 20 patients, also resident in the city of Catania, who had had the onset after 31 December 2004, and who fulfilled the McDonald’s criteria [22] for CIS were enrolled into the study. Furthermore, we have also studied 40 patients with other OND, who had attended our centre during the study period. Among the OND patients 13 (32.5%) were affected by Parkinson’s disease, 12 (30%) by spinocerebellar ataxia, 8 (20%) by amyotrophic lateral sclerosis, three (7.5%) by epilepsy, and each one (2.5%) by trigeminal neuralgia, cervical degenerative disc disease, complete transverse myelitis and myasthenia.

### Sample Size Calculation

Sample size was calculated considering a frequency of the exposure in the general population of 8.8%, average of the frequency of CCSVI among healthy subjects reported in published studies [16]. Considering a 5% level of significance and a power of 80% the minimum number of subjects needed for the study in order to detect an odds ratio (OR) of 2.5 was 185 cases and 185 controls (ratio 1∶1).

### Clinical and Exposure Assessment

All enrolled patients underwent a complete neurological and physical examination including blood pressure measurement, detailed medical history of vascular risks with particular emphasis on venous disease. All neurological examinations were performed by trained and certified examining neurologist (Neurostatus, 2006; available at http://www.neurostatus.net); demographic and clinical data have been also recorded. For each patient the Expanded Disability Status Scale (EDSS) [23] evaluation was obtained the same day ultrasonographic studies were performed.

ECD and TCC ultrasonographies were performed by a single experienced vascular sonographer who attended a course on CCSVI held by Dr Zamboni at the University of Ferrara in 2011. In order to correctly apply the ECD ultrasonograph Zamboni’s criteria for the diagnosis of CCSVI, before the beginning of the study, he also received a further training at the same University.

A GE Vivid E Ultrasound system (GE Healthcare, Horten, Norway), equipped with a 8L-RS (4–12 MHz) linear array transducer was employed for the study of internal jugular vein (IJV) and vertebral veins (VVs) while a 3S-RS Sector Array Probe (1.5–3.6 MHZ) was used for the study of the deep cerebral veins (DCVs). Furthermore a special C-RS Microconvex Ultrasound Probe was used to study internal jugular veins (IJV) under clavear points.

Following the Zamboni’s procedures [7], the exam comprised orthostatic and clinostatic evaluations of both the IJV and vertebral veins (VVs), and the direction of the flow in the internal cerebral vein, the vein of Rosenthal and the vein of Galeno.

The following five parameters were evaluated for each case and control subjects

1. Reflux in the IJV and/or VVs in sitting and supine posture;
2. Reflux in the DCVs;
3. High-resolution B-mode evidence of IJV stenosis;
4. Flow not Doppler detectable in the IJVs and/or VVs;
5. Reverted postural control of the main cerebral venous outflow pathways.

In agreement with literature data, presence of CCSVI was defined as the presence of at least two out of the five parameters [7].

### Blinding Procedures

Each ECD examination was recorded on a DVD and the presence of CCSVI was evaluated by an adjudication panel composed by two expert evaluators (P.F.V. and D.P.L), both unaware of the subjects’ status. Only in case of disagreement between the two experts the sonographer joined the panel to obtain a consensus on CCSVI status.

The panel’s members were blinded to subjects’ status (cases or controls) and unaware of the number of patients with MS, OND, or CIS and controls enrolled in the study. All the members of the adjudication panel attended the course on CCSVI and received a specific training at the University of Ferrara before the study.

To avoid the possible lack of blinding we adopted the following strategies:

1. instructing subjects not to reveal their disease status during Doppler examination;
2. all the enrolled subjects were always placed on the tilt table before the arrival of Doppler evaluator.

### Statistical Analysis

Data were analyzed using STATA 10.0 software packages [24]. Data were double entered into the database. Data cleaning was also performed before the data analysis considering both range and consistence checks.

Quantitative variables were described using mean and standard deviation (m ± SD). The difference between means and the difference between proportions were evaluated by the t-test and the Chi-square test respectively. In case of not a normal distribution appropriate non-parametric tests were performed. Unconditional logistic regression analysis was performed and for each study variable, we calculated OR, 95% confidence interval (CI), and p-value (two-tailed test, p = 0.05). Multivariate analysis was performed to investigate the independent effect of a risk or protective factor after adjustment for one or several other factors or to adjust for confounding variables. Parameters associated with the outcome at the univariate analysis with a threshold of p = 0.10 were included in the model. The model was manually constructed using the likelihood ratio test (LRT) to compare the log-likelihood of the model with and without a specific variable.

Whenever variables were dichotomized or polychotomized, the cut-offs were derived from the pooled distribution of cases and control subjects (e.g., using the median, tertiles, or quartiles).

The possible interaction was also evaluated by the LRT (test of violation of proportional odds). For quantitative exposure the test for linear trend was performed to evaluate the linear or trend effect. Stratified analyses were performed for MS form and disease duration.

Thirty ECD and TCC ultrasonographies have been repeated within one week and Kappa coefficient has been estimated in order to assess intra-rater agreement.

## Results

According to the sample size calculation from the incident cohort of 367 MS patients, we randomly selected 200 MS patients (54%). Out of the 200 randomly selected patients, 40 were not traced, 10 refused, one was bedbound and two were already dead. Finally 148 (74%) MS patients were effectively enrolled in the study. Among the enrolled patients, 105 had relapsing-remitting (RR) MS, 26 had secondary progressive (SP) MS and 17 had primary progressive (PP) MS. No significant differences were found between baseline characteristics (age, gender, age at onset) of MS patients enrolled in the study and the MS patients selected from the incident cohort but not enrolled (n = 52) in the study, except for a longer disease duration recorded among the enrolled patients (175.1±110 versus 126±49.8 months; p = 0.002).

At the end of the study 177 controls have been selected of whom 23 were positive at the screening questionnaire for neurological diseases and underwent a complete neurological examination. Five of them were excluded because presented abnormal neurological examination Finally 172 controls were enrolled. No significant differences were found for age and gender distribution between MS patients and controls. Baseline characteristics of patients affected by MS, CIS, OND and controls are reported in table 1.

**Table 1:** Demographic and clinical characteristics of patients and control subjects.

	CDMS	CIS	OND	Controls
**Patients (n)**	148	20	40	172
**Sex W/M**	93/55	13/7	24/16	100/72
**Age (years)** *	44.3±12.8	38.1±11.6	46.1±13.8	43.0±13.9
**Age at onset (years)** *	31.7±10.3	35.5±10.9	/	/
**Disease duration (months)** *	175.1±110.0	31.7±25.5	/	/

* Values are means ± standard deviation (SD).

CDMS = clinically definite multiple sclerosis;

CIS = clinically isolated syndrome;

OND = other neurological disease;

n = number;

W/M = women/men;

EDSS = Expanded Disability Status Scale.

Intra-rater agreement of TCC-ECD evaluation was good with a kappa value of 0.79 (p<0.001).

CCSVI, defined as the presence of at least two positive venous hemodynamic criteria [7], was present in 28 of the 148 MS patients (18.9%) and in 11 (6.4%) of the 172 controls. CCSVI was further found in 2 (10%) of 20 CIS patients and in 2 (5%) of the 40 OND patients. Table 2 shows the abnormal hemodynamic findings among the different groups. The most common abnormalities in MS were criterion III, high-resolution B-mode evidence of IJV stenosis, followed by criterion II, reflux in the DCVs, criterion V, reverted postural control of the main cerebral venous outflow pathway and criterion I, reflux in the IJV and/or VVs in sitting and supine posture; criterion IV, flow not Doppler detectable in the IJVs and/or VVs, was found in very few patients.

**Table 2:** Distribution of venous hemodynamic criteria among groups.

	CDMS(n = 148)	CIS(n = 20)	OND(n = 40)	Controls(n = 172)
**Criterion I** **n (%)**	17 (11.5%)	3 (15%)	0	6 (3.5%)
**Criterion II** **n (%)**	27 (18.2%)	3 (15%)	0	9 (5.2%)
**Criterion III** **n (%)**	33 (22.3%)	1 (5%)	4 (10%)	13 (7.6%)
**Criterion IV** **n (%)**	4 (2.7%)	0	1 (2.5%)	4 (2.3%)
**Criterion V** **n (%)**	24 (16.2%)	3 (15%)	4 (10%)	21 (12.2%)

Criterion I = Reflux in the IJVs and/or VVs in sitting and supine posture;

Criterion II = Reflux in the DCVs;

Criterion III = High-resolution B-mode evidence of proximal IJV stenoses;

Criterion IV = Flow not Doppler detectable in the IJVs and/or VVs;

Criterion V = Reverted postural control of the main cerebral venous outflow Pathway (Δ CSA);

CDMS = clinically definite multiple sclerosis;

CIS = clinically isolated syndrome;

OND = other neurological disease;

n = number;

IJVs = internal jugular veins;

VVs = vertebral veins;

DCVs = deep cerebral veins.

Considering only the 148 defined MS patients selected from the incidence-cohort and the 172 population-based controls a significant association between MS and CCSVI was found with an OR of 3.41 (95% CI 1.63–7.13; p = 0.001).

**Figure 1:**
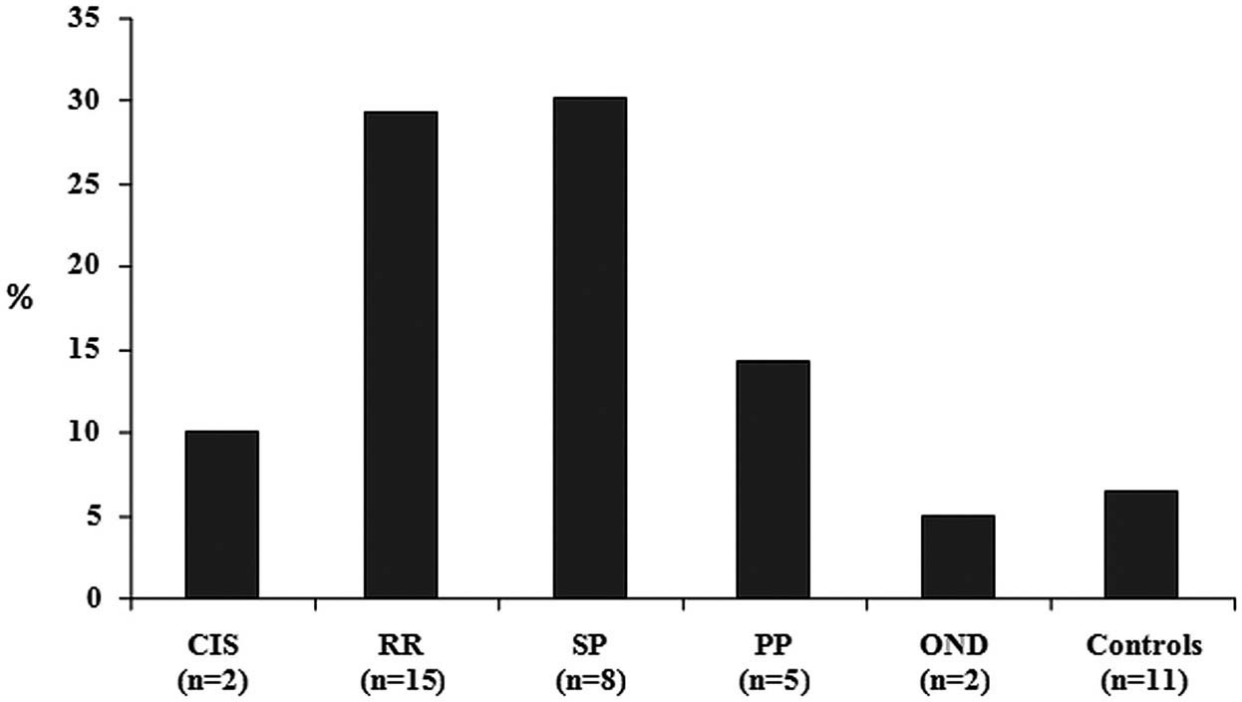
Frequency of CCSVI in different groups. CCSVI = chronic cerebrospinal venous insufficiency; CIS = clinically isolated syndrome; RR = relapsing-remitting; SP = secondary-progressive; PP = primary-progressive; OND = other neurological disease.

Disease duration was dichotomized on the basis of the median value of 144 months; CCSVI was significantly more frequent (p = 0.03) among MS patients with a longer disease duration (18 out of 69, 26.1%) respect to those with a shorter disease duration (10 out of 79, 12.6%). CCSVI was also more frequent among SP and PP patients (30.8% and 29.4% respectively) than among RR MS (14.3%; figure 1) and, as expected, a significantly higher frequency of SP patients presented a longer disease duration (6.3% versus 30.4%; p<0.0001).

When multivariate analysis was stratified considering only the SP and PP forms, a stronger association was found with CCSVI (age adjusted OR = 4.67; 95% CI 1.83–11.9; p = 0.001), while significant but weaker association was found when the analysis was restricted to the RR patients (age adjusted OR = 2.58; 95% CI 1.12–5.92; p = 0.02). We also found a positive, but not significant, association of CCSVI and CIS patients with an age adjusted OR of 2.04 (95% CI 0.40–10.3; p = 0.4).

As shown in table 3, demographic and clinical characteristics between the 28 MS patients with CCSVI and the 120 MS patients without CCSVI differed only for EDSS score which was significantly higher among the CCSVI positive respect to the CCSVI negative MS patients.

**Table 3:** Demographic and clinical findings of patients classified as CCSVI positive (+) and CCSVI negative (−).

	CCSVI^+^(n = 28)	CCSVI^−^(n = 120)	p
**Women n (%)**	17 (60.7%)	76 (63.3%)	0.07
**Age (years)** *	48.0±10.6	45.3±11.3	0.2
**Age at onset (years)** *	32.2±11.1	31.6±10.2	0.7
**Disease duration (months)** *	191±106.6	171.2±110.5	0.4
**EDSS (score)** *	3.5±2.9	1.9±1.9	0.007
**ARR**	0.95	0.87	0.7

* Values are means ± SD;

EDSS = Expanded Disability Status Scale;

ARR = annualized relapse rate.

At the time of the TCC-ECD evaluation, out of the 148 CDMS, 24 (16.2%) did not receive any treatment, 91 (64.5%) were taking Disease-Modifying Drugs (DMD) and 33 (22.3%) Immunosuppressive agents (ISA). In particular among the MS patients with CCSVI, 11 (39.3%) were taking DMD and 12 (42.8%) ISA; on the other hand out of the 120 MS patients without CCSVI, 80 (66.7%) were under DMD treatment and only 21 (17.5%) were under ISA (p = 0.009). However when analysis was stratified according to the MS form not significant differences concerning the type of treatment were found as shown in table 4.

**Table 4:** Treatment and CCSVI. SP/PP MS patients; p = 0.2; RR MS patients; p = 0.4.

	SP/PP (N = 43)				RR (N = 105)			
	*CCSVI +*		*CCSVI −*		*CCSVI +*		*CCSVI −*	
	N	%	N	%	N	%	N	%
**None**	2	15.4	10	33.3	3	20.0	9	10.0
**DMD**	1	7.7	6	20.0	10	6.7	74	82.2
**ISA**	10	76.9	14	46.7	2	13.3	7	7.8
**Total**	**13**	**/**	**30**	**/**	**15**	**/**	**90**	**/**

SP = secondary progressive;

PP = primary progressive;

RR = relapsing remitting;

DMD = disease modifying drugs;

ISA = immunosuppressive agents.

## Discussion

In our study we found a significant association between CCSVI and MS. Differences were evident among the groups studied: MS patients showed the highest CCSVI prevalence compared to normal subjects and OND patients. Furthermore CCSVI was more frequent in patients with progressive MS than in patients with RRMS or CIS and in patients with longer disease duration.

During the last few years, several studies have been carried out to evaluate the possible association between MS and CCSVI and most of them have reported a higher frequency of CCSVI among MS patients, suggesting a possible pathogenetic role [7], [16]. However a great variation of the frequency of CCSVI among both MS patients and controls has been reported. According to a recent systematic review the average prevalence of CCSVI is 55.6% among MS patients (ranging from 100% to 0%) and 11.4% among the control subjects (ranging from 0 to 35.7%) [16]. Due to this large variability, probably related to the diagnosis of CCSVI, to date, there is no general consensus about the exact frequency of CCSVI in MS and about its putative pathogenetic role [7], [11], [13], [14], [16].

To the best of our knowledge, this study represents the first population-based case control-study carried out to evaluate possible association between CCSVI and MS. Frequency of CCSVI, in fact, has been estimated in a randomly selected sample of a population-based incident cohort of MS patients, thus reducing the risk of a possible selection bias among cases and allowing also the generalizability of the obtained results. Nevertheless the choice of appropriate control subjects is probably the most challenging aspect in case–control studies, and, as it is well known, several strategies should be adopted for selection in order to obtain a representative control population avoiding selection bias. To this reason, to obtain a representative sample of the general population, controls were randomly selected from the GPs roster and the GPs involved in the study were selected from the 10 municipalities of the town. Furthermore, in order to avoid a possible observer bias, great attention has been made to guarantee the blinding procedure.

In agreement with literature data, we observed an higher frequency of CCSVI among MS patients respect to the control population. However in our population-based study, CCSVI was significantly more frequent among MS patients with a longer disease duration respect to those with a shorter disease duration. This observation seems to be supported by the higher frequency of CCSVI among the SP patients, and consequently with a longer disease duration, respect to the RR MS [7], [25]. These results are in agreement with recently published studies that have also found a higher prevalence of CCSVI only in more advanced disease [25], [26], [27] and in a limited proportion of patients with CIS [26], [28], supporting the hypothesis that CCSVI could be subsequent to the MS onset [25]. Moreover, the higher frequency of ISA therapy found among CCSVI positive MS patients could be explained by finding higher frequency of CCSVI among SP MS patients who were more frequently treated with ISA therapy.

Nevertheless the prevalence of CCSVI found in our sample is far from that initially reported by Zamboni et colleagues [7], as well as from other published data [11], [12], [25], [29], [30]. A possible source of variability is related to the accuracy and reliability of the ECD in detecting CCSVI, even if, in the majority of the published studies, ECD was performed according to the Zamboni’s procedure [7], [31]. To this reason, in order to obtain more comparable data, all the ECD in our study were performed by a single experienced vascular sonographer who attended a course on CCSVI held by Dr Zamboni, and a good reliability (k = 0.79) has been obtained when a random sample of ECDs were repeated. Furthermore, in order to avoid a possible observer bias, both the sonographer and the members of the adjudication panel were unaware on the subjects’ status.

Another important point to take into account is related to the retrospective nature of the study that did not allow us to establish the exact sequence of the events. To this reason we are not able to be certain that CCSVI exposure occurred before or after the disease onset. Our results indicate that only 18.9% of patients with MS, and 10% with CIS presented with CCSVI. We further found CCSVI to be significantly more frequent among MS patients with longer disease duration, and in patients with the progressive forms of MS. According to these observations, CCSVI could be subsequent to the disease onset, making impossible to exclude a possible “reverse causality”. Even if a “causative relationship” between CCSVI and MS has been suggested, it should be underlined that association not necessary imply causation and that observational study alone does not allow us to establish a cause-effect relationship. To the best of our knowledge all the data available in literature concerning the relationship between CCSVI and MS come from retrospective case-control studies that are unable to determine whether the exposure precede the outcome (temporal relationship), the only absolutely essential or necessary criterion of causality [32]. We cannot exclude, in fact, either the possibility that CCSVI is in the causal pathway of MS, or a possible indirect association between CCSVI and MS, whereby they are mediated by related but not direct pathways. A recent study, carried out to evaluate the association between CCSVI and environmental factors, in a large volunteer control group without known central nervous system pathology, has highlighted a possible association between CCSVI and history of infectious mononucleosis [33]. Epstein-Barr virus (EBV) infection is one of the most important potential risk for the development of MS [34], and there are also some evidences that EBV infection can act as a precipitating factor for venous thrombo-embolism in immunocompromised patients [33]. Whether EBV infection may damage the venous endothelium, causing venous thromboses and strictures in the cranial and spinal venous drainage system, is unknown yet and further longitudinal studies should investigate the relationship between CCSVI and EBV infection. Moreover, it has been demonstrated that MS patients have non-specific alterations of cerebral venous drainage and they share similar abnormalities with chronic migraineurs [35].

Proximal IJV stenosis followed by reflux in deep cerebral veins, reverted postural control of the main cerebral venous outflow pathways and reflux in IJVs and/or VVs in both supine and upright position were more frequently found in MS patients than in CIS and OND patients and in controls. Similar findings with different percentages were found by other groups [7], [11], [25], [29], [30], [31], [36], [37]. A recent post mortem study demonstrated higher frequency of intraluminal IJVs and azygos abnormalities in MS patients than in controls, probably explaining the reported higher frequency of IJV stenosis [38].

Whether or not venous abnormalities can contribute to the severity of the disease is questionable. However, our results, in agreement with literature data [7], [11], [25], [26], [27], [29], [30], suggest that disability, longer disease duration and progressive forms could partly account for the existence of CCSVI in MS patients. It is conceivable that, as reported by other studies [39]–[41], hypoperfusion of the brain parenchima in advanced MS patients could partly drive the burden of disability.

In conclusion, even if the suggested causal-relationship could be supported by the positive associations reported in different studies, partially fulfilling the consistency criterion [32], several factors limit the interpretation of the results. Indeed, the extra-cranial venous system is highly variable with different collateral pathways and position-dependent flow changes [9], [15]. Further studies in healthy subjects of different age are needed to better understand the physiological variability as well as the factors which could have an influence on the venous system.

Moreover it has to be kept in mind that DMD and ISA treatment may also have an influence on the venous system and the results of the ultrasound investigation which should be addressed in further studies.

From our point of view, at the state of the art, results from observational studies must be interpreted with caution and longitudinal multicentre studies on large cohort of CIS should be performed to better understand the correlation between CCSVI and progression.

## References

[1] Charcot JM (1868) Histology of “sclerose en plaque”. Gazette Hosp Paris 41: 554–565.

[2] Fog T (1965) The topography of plaques in multiple sclerosis with special reference to cerebral plaques. Acta Neurol Scand. Suppl 151–167. 5213727

[3] Schelling P (1986) Demanding venous reflux into the skull of spine: relevance to multiple sclerosis. Med Hypotheses 2: 141–148. 10.1016/0306-9877(86)90003-4 3641027

[4] Tan IL, Van Schijndel RA, Pouwels PJ, Van Walderveen MA, Reichenbach JR, et al (2000) MR venography of multiple sclerosis. Am J Neuroradiol 21: 1033–1042. PMC7973892 10871010

[5] Adams CWM (1989) Vascular aspects of multiple sclerosis. In: A Colour Atlas of Multiple Sclerosis & Other Myelin Disorders. London: Wolfe Medical Publication. 184–187.

[6] Zamboni P (2006) Iron-dependent inflammation in venous disease and proposed parallels in multiple sclerosis. J R Soc Med 96: 589–593. 10.1258/jrsm.99.11.589 PMC1633548 17082306

[7] Zamboni P, Galeotti R, Menegatti E, Malagoni AM, Tacconi G, et al (2009) Chronic cerebrospinal venous insufficiency in patients with multiple sclerosis. J Neurol Neurosurg Psychiatry 80: 392–399. 19060024 10.1136/jnnp.2008.157164 PMC2647682

[8] Zamboni P, Menegatti E, Galeotti R, Malagoni AM, Tacconi G, et al (2009) The value of cerebral Doppler venous hemodynamics in the assessment of multiple sclerosis. J Neurol Sci 282: 21–27. 19144359 10.1016/j.jns.2008.11.027

[9] Stolz E, Kaps M, Kern A, Babacan SS, Dorndorf W (1989) Transcranial color-coded duplex sonography of intracranial veins and sinuses. Reference data from 130 volunteers. Stroke 90: 1070–1075. 10.1161/01.str.30.5.1070 10229746

[10] Zamboni P, Menegatti E, Bartolomei I, Galeotti R, Malagoni AM, et al (2007) Intracranial venous hemodynamics in multiple sclerosis. Curr Neurovasc Res 4: 252–258. 18045150 10.2174/156720207782446298

[11] Krogias C, Schröder A, Wiendl H, Hohlfeld R, Gold R (2010) “Chronic cerebrospinal venous insufficiency” and multiple sclerosis: critical analysis and first observation in an unselected cohort of MS patients. Nervenartz 81: 740–746. 10.1007/s00115-010-2972-1 20386873

[12] Zivadinov R, Lopez-Soriano A, Weinstock-Guttman B, Schirda CV, Magnano CR, et al (2011) Use of MR venography for characterization of the extracranial venous system in patients with multiple sclerosis and healthy control subjects. Radiology 258: 562–570. 21177394 10.1148/radiol.10101387

[13] Doepp F, Paul F, Valdueza JM, Schmierer K, Schreiber SJ (2010) No cerebrovascular venous congestion in patients with multiple sclerosis. Ann Neurol 68: 173–180. 20695010 10.1002/ana.22085

[14] Sundström P, Wåhlin A, Ambarki K, Birgander R, Eklund A, et al (2010) Venous and cerebrospinal fluid flow in multiple sclerosis: a case-control study. Ann Neurol 68: 255–259. 20695018 10.1002/ana.22132

[15] Wattjes MP, Van Oosten BW, De Graaf WL, Seewann A, Bot JC, et al (2010) No association of abnormal cranial venous drainage with multiple sclerosis: a magnetic resonance venography and flow-quantification study. J Neurol Neurosurg Psychiatry 82: 429–435. 20980483 10.1136/jnnp.2010.223479

[16] Laupacis A, Lillie E, Dueck A, Straus S, Perrier L, et al (2011) Association between chronic cerebrospinal venous insufficiency and multiple sclerosis: a meta-analysis. CMAJ 183: 1203–1212. 10.1503/cmaj.111074 PMC3216446 21969411

[17] Nicoletti A, Patti F, Lo Fermo S, Sorbello V, Reggio E, et al (2005) Possible increasing risk of multiple sclerosis. Neurology 65: 1259–1263. 16247054 10.1212/01.wnl.0000180628.38786.85

[18] Nicoletti A, Patti F, Lo Fermo S, Messina S, Bruno E, et al (2011) Increasing frequency of multiple sclerosis in Catania, Sicily: a 30-year survey. Mult Scler 17: 273–280. 21071466 10.1177/1352458510386995

[19] Poser CM, Paty DW, Scheinberg L, McDonald WI, Davis FA, et al (1983) New diagnostic criteria for multiple sclerosis: guidelines for research protocols. Ann Neurol 13: 227–231. 6847134 10.1002/ana.410130302

[20] Lublin FD, Reingold SC (1996) Defining the clinical course of multiple sclerosis: results of an international survey: National Multiple Sclerosis Society (USA) Advisory Committee on Clinical Trials of New Agents in Multiple Sclerosis. Neurology 46: 907–911. 8780061 10.1212/wnl.46.4.907

[21] Meneghini F, Rocca WA, Anderson DW, Grigoletto F, Morgante L, et al (1992) Validating screening instruments for neuroepidemiologic surveys: experience in Sicily. Sicilian Neuro-Epidemiologic Study (SNES) Group. J Clin Epidemiol 45: 319–331. 1314889 10.1016/0895-4356(92)90033-j

[22] McDonald WI, Compston A, Edan G, Goodkin D, Hartung HP, et al (2001) Recommended diagnostic criteria for multiple sclerosis: guidelines from the international panel on the diagnosis of multiple sclerosis. Ann Neurol 51: 121–127. 10.1002/ana.1032 11456302

[23] Kurtzke JF (1983) Rating neurologic impairment in multiple sclerosis: an expanded disability status scale (EDSS). Neurology 33: 1444–1452. 6685237 10.1212/wnl.33.11.1444

[24] STATA Corp. STATA statistical software: release 10.0. College Station, TX: STATA Corporation.

[25] Zivadinov R, Marr K, Cutter G, Ramanathan M, Benedict RH, et al (2011) Prevalence, sensitivity, and specificity of chronic cerebrospinal venous insufficiency in MS. Neurology 77: 138–144. 21490322 10.1212/WNL.0b013e318212a901

[26] Yamout B, Herlopian A, Issa Z, Habib RH, Fawaz A, et al (2010) Extracranial venous stenosis is an unlikely cause of multiple sclerosis. Mult Scler 16: 1341–1348. 21041329 10.1177/1352458510385268

[27] Bastianello S, Romani A, Viselner G, Tibaldi EC, Giugni E, et al (2011) Chronic cerebrospinal venous insufficiency in multiple sclerosis: clinical correlates from a multicentre study. BMC Neurology 11: 132. 22029656 10.1186/1471-2377-11-132 PMC3221625

[28] Baracchini C, Perini P, Calabrese M, Causin F, Rinaldi F, et al (2011) No evidence of chronic cerebrospinal venous insufficiency at multiple sclerosis onset. Ann Neurol 69: 90–99. 21280079 10.1002/ana.22228

[29] Al-Omari MH, Rousan LA (2010) Internal jugular vein morphology and hemodynamics in patients with multiple sclerosis. Int Angiol 29: 115–120. 20351667

[30] Simka M, Kostecki J, Zaniewski M, Majewski E, Hartel M (2010) Extracranial Doppler sonographic criteria of chronic cerebrospinal venous insufficiency in the patients with multiple sclerosis. Int Angiol 29: 109–114. 20351666

[31] Menegatti E, Genova V, Tessari M, Malagoni AM, Bartolomei I, et al (2010) The reproducibility of colour Doppler in chronic cerebrospinal venous insufficiency associated with multiple sclerosis. Int Angiol 29: 121–126. 20351668

[32] Hill BA (1965) The environment and disease: Association or causation? Proceedings of the Royal Society of Medicine 58: 295–300. 14283879 10.1177/003591576505800503 PMC1898525

[33] Dolic K, Weinstock-Guttman B, Marr K, Valnarov V, Carl E, et al. (2011) Risk factors for chronic cerebrospinal venous insufficiency (CCSVI) in a large cohort of volunteers. PLoS One 6: 11. Available: doi:10.1371/journal.pone.0028062 Accessed 30 Nov 2011. 10.1371/journal.pone.0028062 PMC3227626 22140507

[34] Santiago O, Gutierrez J, Sorlozano A, De Dios Luna J, Villegas E, et al (2010) Relation between Epstein-Barr virus and multiple sclerosis: analytic study of scientific production. Eur J Clin Microbiol Infect Dis 29: 857–866. 20428908 10.1007/s10096-010-0940-0

[35] Ertl-Wagner B, Koerte I, Kuempfel T, Blaschek A, Laubender RP, et al (2011) Non-specific alterations of craniocervical venous drainage in multiple sclerosis revealed by cardiac-gated phase-contrast MRI. MSJ. doi 10.1177/1352458511432742. 10.1177/1352458511432742 22194216

[36] Doepp F, Würfel JT, Pfueller CF, Valdueza JM, Petersen D, et al (2011) Venous drainage in multiple sclerosis: A combined MRI and ultrasound study. Neurology 77(19): 1745–1751. 22031530 10.1212/WNL.0b013e318236f0ea

[37] Tsivgoulis G, Mantatzis M, Bogiatzi C, Vadikolias K, Voumvourakis K, et al (2011) Extracranial venous hemodynamics in multiple sclerosis. Neurology 77: 1241–1245. 21849653 10.1212/WNL.0b013e318230a149

[38] Diaconu C, Staugaitis S, McBride J, Schwanger C, Rae-Grant A, et al. (2011) Anatomical and histological analysis of venous structures associated with chronic cerebro-spinal venous insufficiency. Mult Scler 17: S9–S52. Presented at 5th joint triennial congress of the ECTRIMS and ACTRIMS, Amsterdam 19–22 Oct 2011.

[39] De Keyser J, Steen C, Mostert JP, Koch MW (2008) Hypoperfusion of the cerebral white matter in multiple sclerosis: possible mechanisms and pathophysiological significance. J Cereb Blood Flow Metab 28: 1645–1651. 18594554 10.1038/jcbfm.2008.72

[40] Varga AW, Johnson G, Babb JS, Herbert J, Grossman RI, et al (2009) White matter hemodynamic abnormalities precede sub-cortical gray matter changes in multiple sclerosis. J Neurol Sci 282: 28–33. 19181347 10.1016/j.jns.2008.12.036 PMC2737614

[41] Wuerfel J, Paul F, Zipp F (2007) Cerebral blood perfusion changes in multiple sclerosis. J Neurol Sci 259: 16–20. 17382348 10.1016/j.jns.2007.02.011

